# Chromosome Painting Provides Insights Into the Genome Structure and Evolution of Sugarcane

**DOI:** 10.3389/fpls.2021.731664

**Published:** 2021-08-27

**Authors:** Zhuang Meng, Qinnan Wang, Haris Khurshid, Ghulam Raza, Jinlei Han, Baohua Wang, Kai Wang

**Affiliations:** ^1^School of Life Sciences, Nantong University, Nantong, China; ^2^Key Laboratory of Genetics, Breeding and Multiple Utilization of Crops (MOE), Fujian Agriculture and Forestry University, Fuzhou, China; ^3^Institute of Bioengineering, Guangdong Academy of Sciences, Guangzhou, China; ^4^Oilseeds Research Program, National Agricultural Research Centre, Islamabad, Pakistan; ^5^Agricultural Biotechnology Division, National Institute for Biotechnology and Genetic Engineering, Faisalabad, Pakistan

**Keywords:** *Saccharum*, chromosome painting, FISH, ploidy, interspecies recombination

## Abstract

The genus *Saccharum* is composed of species with high polyploidy and highly varied chromosome numbers, laying a challenge for uncovering its genomic structure and evolution. We developed a chromosome 2 painting (CP2) probe by designing oligonucleotides covering chromosome 2 of *Saccharum spontaneum* (2n = 8x = 64). Fluorescence *in situ* hybridization (FISH) using this CP2 probe revealed six types of ploidies from twenty *S. spontaneum* clones, including 6x, 8x, 10x, 11x, 12x, and 13x clones. The finding of *S. spontaneum* clones with uneven of ploid suggested that certain *S. spontaneum* clones come from hybridization. It renews our knowledge that *S. spontaneum* is derived from autopolyploidization. Combined with a *S. spontaneum*-specific probe, chromosome 2-derived chromosome or fragments from either *S. spontaneum* or *Saccharum officinarum* can be identified in sugarcane modern cultivars. We revealed unexpected high level of interspecific recombination from introgressive *S. spontaneum* chromosomes (>50.0%) in cultivars ROC22 and ZZ1, indicating frequent chromosome exchange in cultivars. Intriguingly, we observed interspecific recombination recurring among either homoeologous or non-homoeologous chromosomes in sugarcane cultivars. These results demonstrated that chromosome painting FISH is a powerful tool in the genome dissection of sugarcane and provide new insights into the genome structure and evolution of the complex genus *Saccharum*.

## Introduction

Chromosome painting (CP) is a technique to visualize the entire chromosome via fluorescence *in situ* hybridization (FISH) using chromosome-specific painting probes (Pinkel et al., 1988). CP has been verified as a powerful tool for diagnosing chromosome abnormalities, investigating karyotypic alterations during evolution, and constructing ancestral karyotypes (Natarajan et al., 1992; Willem et al., 2006; Graphodatsky et al., 2011; Marshall and Obe, 2015). In the past few decades, CP probes have mainly been amplified from flow-sorted or microdissected chromosomes followed by degenerate oligonucleotide-primed PCR amplification (Cremer et al., 1988; Yang et al., 1999; Ferguson-Smith and Trifonov, 2007), and they have been successfully applied to chromosomes of more than 40 mammalian species, such as humans, birds, and insects (Cremer et al., 1988; Zimmer et al., 1997; Fuchs et al., 1998).

In plants, CP probes derived from flow-sorted or microdissected chromosomes barely yield satisfactory and reproducible results (Fuchs et al., 1996). A major cause is the prevalence of repetitive DNAs in the genomes, which results in unfavorable non-specific hybridization signals. To overcome this problem, CP based on large insert DNA clone (YAC/BAC) probes with low amounts of repetitive sequences has been developed and successfully applied in studies of the genomic structure and evolution of plants (Fransz et al., 2000; Xiaomin et al., 2008; Dóra et al., 2010; Kai et al., 2010; Mandakova et al., 2010; Peters et al., 2012). However, in plants, especially those with large and complex genomes, it was almost impossible to screen entire chromosome-covered BACs without or with low levels of repetitive sequences. Instead of YAC- or BAC-based CP probes, single-copy gene-based CP probes were successfully employed in *Cucumis sativus* (Lou et al., 2014). However, PCR amplification of single-copy genes from the entire chromosome is also labor-intensive and time consuming.

Technical advances in DNA synthesis have made it possible to massively synthesize oligonucleotides (oligos) designed based on genome assembly without repetitive sequences. FISH studies in plants have shown the superior resolution and versatility of oligo-based probes compared to conventional genomic clone- or single-copy gene-based probes (Han et al., 2015; Qu et al., 2017; Braz et al., 2018; He et al., 2018; Hou et al., 2018; Meng et al., 2018, 2020; Xin et al., 2018; Liu et al., 2019; Simonikova et al., 2019). Recently, a *k*-mer analysis-based protocol was developed to generate whole-genome paints with excellent specificity in maize (Albert et al., 2019), which in turn confers the applicability of oligo-based CP in plants with high repetitive sequence content (Zhang et al., 2021).

Sugarcane is one of the most important sugar and biofuel crops in the world, providing 80% of the world’s sugar and 40% of its ethanol. According to conventional taxonomy, the genus *Saccharum* typically includes six species, namely, *Saccharum officinarum*, *Saccharum sinense*, *Saccharum barberi*, *Saccharum edule*, *Saccharum robustum*, and *S. spontaneum*. Among the six species, *S. spontaneum* and *S. robustum* are the only wild species (Irvine, 1999). All of the *Saccharum* species are polyploid with highly variable chromosomal numbers (Panje and Babu, 1960; Ming et al., 2010) (2n = 40–128), which caused the genome structure and evolution studies in the genus to lag behind those of other plants. Recently, the whole-genome sequences of *S. spontaneum* (*x* = 8) (Zhang et al., 2018) and modern cultivar (Garsmeur et al., 2018) have been achieved, which pave the way for the application of oligo-based CP in sugarcane. In this study, we developed the first whole chromosome painting probe in sugarcane. By FISH assays, we revealed ploidy diversity in clone of the wild species *S. spontaneum*, suggesting a non-autopolyploidization origin in certain *S. spontaneum* clones. Combined with a *S. spontaneum*-specific probe, we can trace specific chromosomes or fragments in complex modern cultivars, demonstrating that this approach is a powerful strategy for precise dissection of the genome structure in cultivars.

## Materials and Methods

### Plant Materials

Twenty-two *S. spontaneum* clones (Np-X, 2012-46, SES208, Yunnan84-268, Yunnan82-16, Yunnan82-67, Yunnan82-106, Yunnan82-29, Yunnan82-110, Yunnan76-III-13, Yunnan76-III-18, Sichuan92-42, Sichuan79-II-20, Sichuan79-II-18, Sichuan88-16, Sichuan79-I-1, Sichuan79-II-11, Guangdong30, Fujian89-I-17, Fujian87-I-4, Fujian89-I-19, and Guizhou78-II-28), two *S. officinarum* clones (LA Purple and Badila), *S. robustum* 51NG63 and two modern cultivars (ROC22 and ZZ1) were used in this study. All of the plants were grown in the greenhouse at Fujian Agriculture and Forestry University with a 16 h light/8 h dark photoperiod at 30°C.

### Design and Synthesis of Oligo-Based Chromosome 2 Painting Probe

The oligo-based painting probe of *S. spontaneum* chromosome 2 (Supplementary Data Sheet 1) was designed using Chorus software^1^ as previously described (Han et al., 2015). Briefly, oligos (59 nt) specific to chromosome 2, based on the *S. spontaneum* AP85-441 genome^2^ (Zhang et al., 2018), were selected throughout the chromosome 2 pseudomolecule. A total of 33,975 chromosome 2-specific oligos were selected to cover the entire chromosome (114 Mb). The oligos were synthesized *de novo* in parallel by MY microarray (Ann Arbor, MI, United States). Labeling of the chromosome painting probe was performed according to a published protocol (Han et al., 2015).

### Chromosome Spread Preparation

Chromosome spreads were prepared as previously described (Meng et al., 2018) with several modifications. Briefly, root tips were harvested from sugarcane and treated in nitrous oxide at a pressure of 10.9 atm (∼160 psi) for 1–2 h, fixed in Carnoy’s fixative (3 ethanol:1 acetic acid) and stored at −20°C until use. Subsequently, the root tips were digested in an enzymatic solution with 2% cellulase (Yakult Pharmaceutical, Tokyo, Japan) and 1% pectolyase (Sigma Chemical, St. Louis, MO, United States) at 37°C for 1 h and then squashed with a cover slip. After the slides were frozen in liquid nitrogen, the cover slips were removed, and the slides were dehydrated with an ethanol series (70, 90, and 100%, 5 min each) prior to FISH assay.

### FISH Assays Using Oligo and rDNA Probes

The biotin- or digoxigenin-labeled chromosome 2 painting probe synthesized from the oligo pool was directly used for FISH. Rice 45S and 5S rDNAs (Gong et al., 2002) were labeled with either digoxigenin-11-dUTP (Roche Diagnostics, United States) or biotin-16-dUTP (Roche Diagnostics, United States) using standard nick translation reactions. FISH was performed following published protocols (Meng et al., 2018). First, the hybridization mixture (50% formamide, 10% dextran sulfate, 20x SSC, 50 ng labeled probe) was denatured at 90°C for 5 min before being applied to the denatured chromosome slides. Afterward, the chromosome slides were denatured in 70% formamide in 2x SSC at 70°C for 1 min and dehydrated in an ethanol series (70, 90, and 100%; 5 min each). Next, the hybridization mixture was applied to the denatured chromosome slides and incubated for 12 h at 37°C. Then, the slides were washed in 2x SSC, 50% formamide in 2x SSC, and in 2x SSC at 42°C for 5 min each. Subsequently, digoxigenin- and biotin-labeled probes were detected using rhodamine-conjugated anti-digoxigenin (Roche Diagnostics, United States) and fluorescein-conjugated avidin (Life Technologies, United States), respectively. Chromosome slides were counterstained with 4′, 6′-diamidino-phenylindole (DAPI) in a VectaShield antifade solution (Vector Laboratories, Inc., Burlingame, CA, United States). FISH signals were detected under an Olympus BX63 fluorescence microscope. Images were captured and merged by cellSens Dimension 1.9 software with an Olympus DP80 CCD camera. For image assay, 7–10 cells were analyzed. The final images were processed and adjusted by Adobe Photoshop CC software.

## Results

### Development of Individual Chromosome Painting Probes for *S. spontaneum*

**FIGURE 1:**
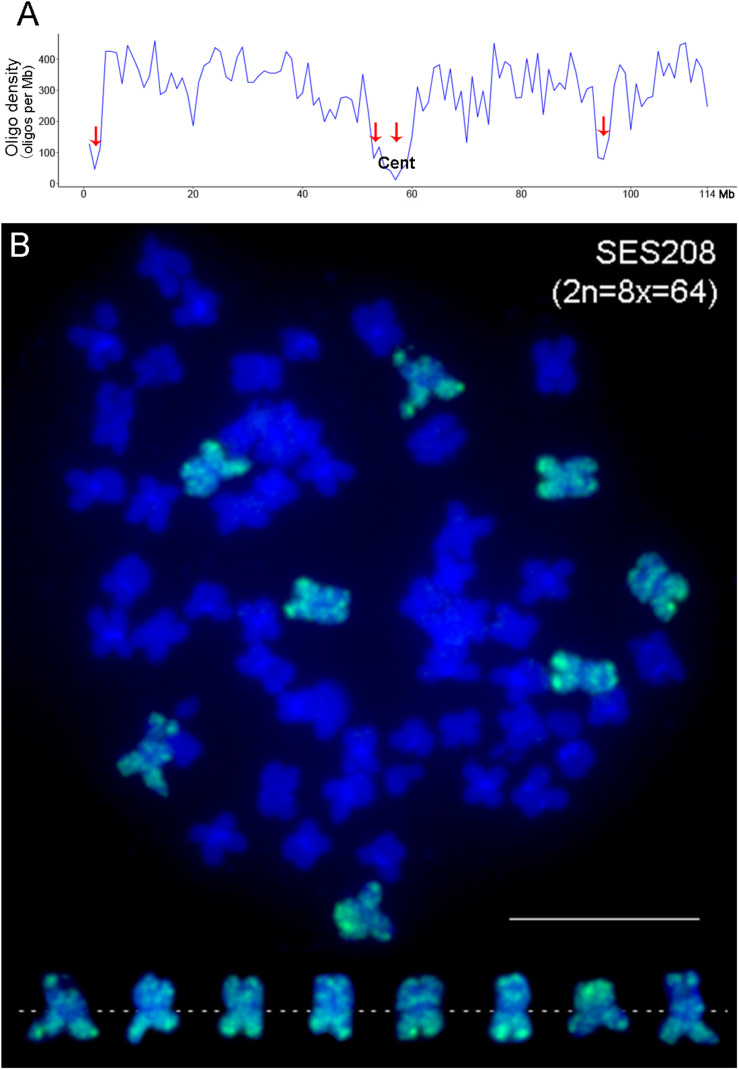
Development of oligo-based CP2 probe in *S. spontaneum*. **(A)** The distribution of oligos on chromosome 2. The *x*- and *y*-axis indicate the position on chromosome 2 and the oligo density (oligo numbers per megabase window), respectively. The red arrows indicate the genomic regions containing a low density of oligos. **(B)** FISH mapping of the CP2 probe in *S. spontaneum* SES208 (2n = 8x = 64). The eight copies of chromosome 2 were digitally excised from the same cell and are shown in the bottom. Scale bar, 10 μm.

We developed an oligo-based chromosome 2 painting (CP2) probe based on the genome assembly of *S. spontaneum* AP85-441 (*x* = 8) chromosome 2 (Zhang et al., 2018). A total of 33,975 oligos (59 nucleotides in size) distributed throughout 114 megabase (Mb) chromosome 2 were designed according to a previously developed pipeline (Han et al., 2015). The CP2 probe had an average density of 298 oligos per Mb. Four regions, i.e., 1–2 Mb, 52–53 Mb, 54–59 Mb, and 93–95 Mb, were enriched with repetitive sequences and thus contained obviously fewer oligos (Figure 1A, arrows). As expected, the centromeric region, i.e., 54–59 Mb (Zhang et al., 2017, 2018), demonstrated the lowest oligo density (Figure 1A).

The designed oligos were then synthesized and labeled as a FISH probe. When hybridized to metaphase chromosomes of the octoploid *S. spontaneum* SES208 (2n = 8x = 64), we observed strong signals on the eight copies of chromosome 2 (Figure 1B). No unspecific signals were detectable from any other chromosomes, indicating that these oligos were specific to chromosome 2 and could potentially be used as reliable markers for chromosome 2 identification in the highly polyploid species *S. spontaneum*.

### Chromosome Painting Is a Reliable Tool for Ploidy Identification in *S. spontaneum*

**FIGURE 2:**
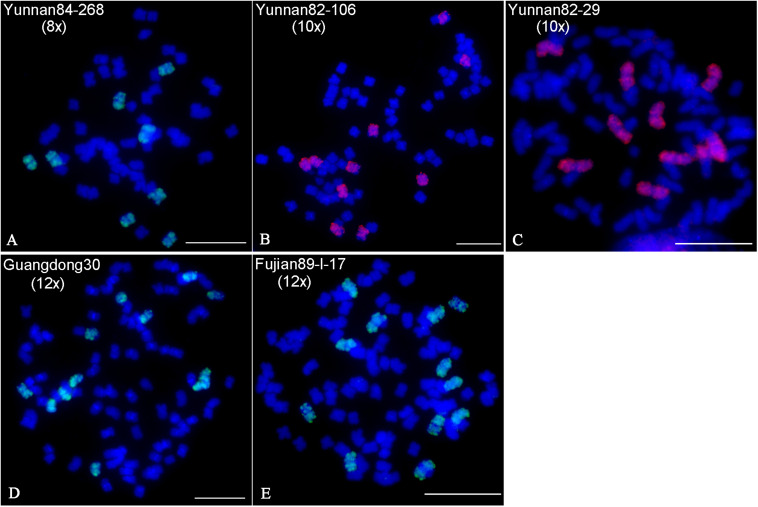
Identification of ploidies in *S. spontaneum* clones by chromosome painting. The CP2 probe was hybridized to the mitotic metaphase chromosomes of five *S. spontaneum* clones, Yunnan84-268 **(A)**, Yunnan82-106 **(B)**, Yunnan82-29 **(C)**, Guangdong30 **(D)**, and Fujian89-I-17 **(E)**. Scale bars, 10 μm.

*Saccharum spontaneum* shows the highest level of genetic diversity in the genus *Saccharum*, with nearly 40 chromosome-number types (2n = 40–128) (Panje and Babu, 1960; Meng et al., 2020). Due to large chromosome numbers, accurate identification of ploidy in *S. spontaneum* varieties has been intractable. We tentatively performed FISH using the CP2 probe in five *S. spontaneum* clones, i.e., Yunnan84-268, Yunnan82-106, Yunnan82-29, Guangdong30, and Fujian89-I-17. As a result, we obtained different copy numbers of chromosome 2 by detecting chromosome painting signals: eight in Yunnan84-268, ten in both Yunnan82-106 and Yunnan82-29, and twelve in both Guangdong30 and Fujian89-I-1 (Figures 2A–E). Therefore, we can readily determine the ploidy of the tested clones, i.e., octaploid for Yunnan84-268, decaploid for Yunnan82-106 and Yunnan82-29, and dodecaploid for Guangdong30 and Fujian89-I-17 (Figure 2).

**FIGURE 3:**
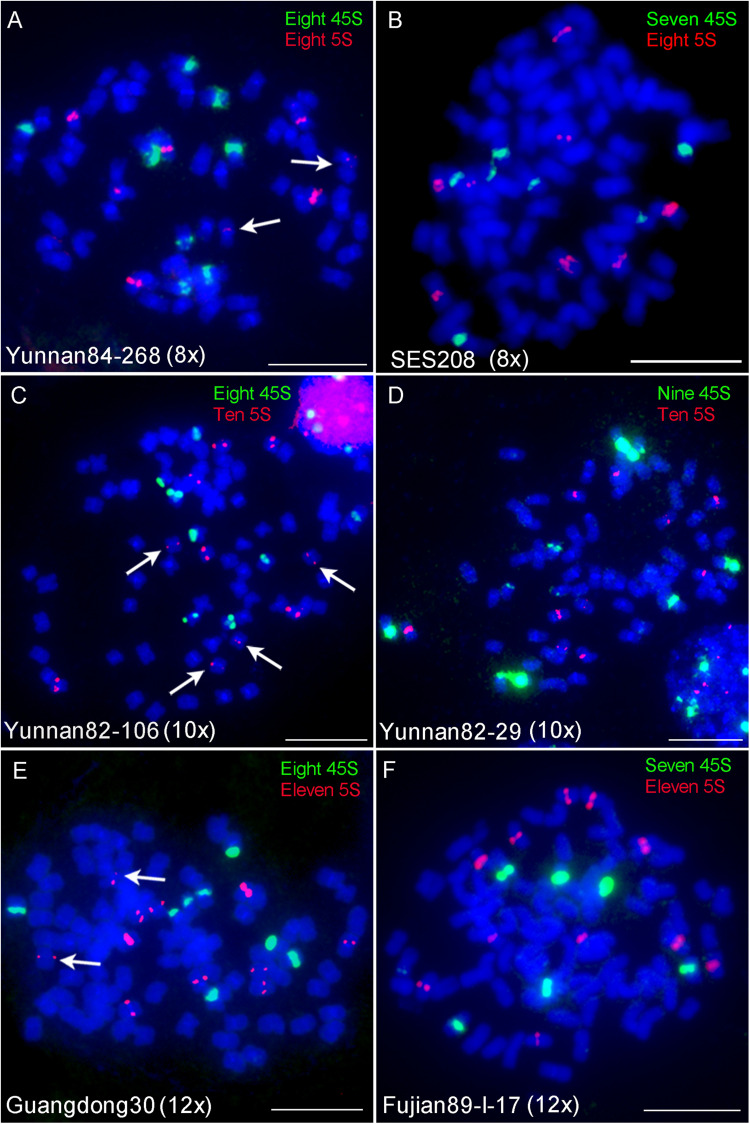
FISH mapping of 45S and 5S rDNAs in *S. spontaneum*. Dual-color FISH assay using probe pairs of 45S (green) and 5S rDNA (red) in six *S. spontaneum* clones Yunnan84-268 (octaploid) **(A)**, SES208 (octaploid) **(B)**, Yunnan82-106 (decaploid) **(C)**, Yunnan82-29 (decaploid) **(D)**, Guangdong30 (dodecaploid) **(E)**, and Fujian89-I-17 (dodecaploid) **(F)**. The signal numbers of each probe are displayed. Arrows indicate the weak signals. Scale bars, 10 μm.

Fluorescence *in situ* hybridization using 45S or 5S rDNA probes has been used to indicate ploidy levels previously in sugarcane (Jenkin et al., 1995; Cuadrado et al., 2004). To test the reliability of the 45S and 5S rDNA strategy, we performed FISH using 45S or 5S rDNA probes in six *S. spontaneum* clones, including two octaploids Yunnan84-268 and SES208, two decaploids Yunnan82-106 and Yunnan82-29, and two dodecaploids Guangdong30 and Fujian89-I-17. The results showed eight 45S and 5S rDNA signals in Yunnan84-268 (octoploid), seven 45S and eight 5S signals in SES208, eight 45S and ten 5S signals in Yunnan82-106 (decaploid), nine 45S and ten 5S signals in Yunnan82-29 (decaploid), eight 45S and eleven 5S signals in Guangdong30 (dodecaploid), and seven 45S and eleven 5S signals in Fujian89-I-17 (dodecaploid) (Figures 3A–F). The locus numbers of either 45S or 5S rDNA varied in clones even with the same ploidy. Moreover, both 45S and 5S rDNAs showed high variations in signal intensities, and some were too weak to be detectable readily (arrows in Figure 3). Taken together, these results indicate that neither 45S nor 5S rDNA is a reliable indicator of ploidy in *S. spontaneum*.

### Non-autopolyploidization Origin Revealed by Chromosome Painting Assay in *S. spontaneum*

**FIGURE 4:**
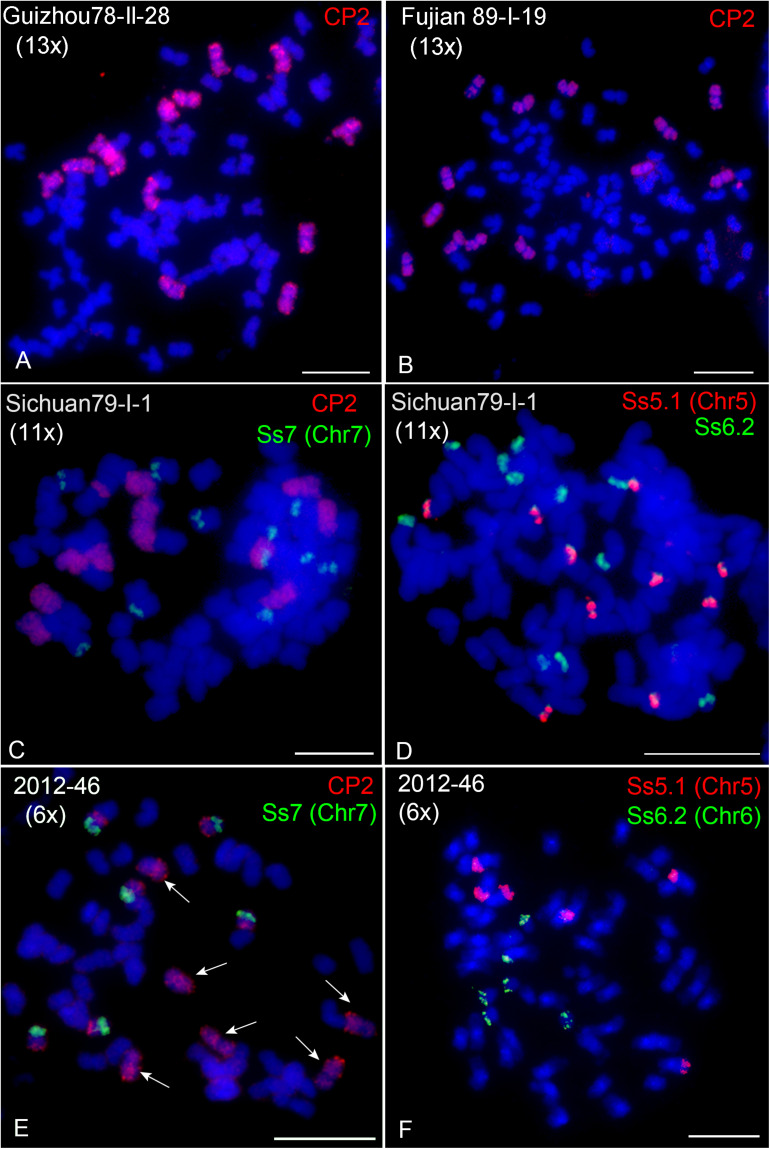
FISH mapping in hexaploid, hendecaploid, and tridecaploid *S. spontaneum* clones. **(A,B)** CP2-FISH mapping in Guizhou78-II-28 **(A)** and Fujian89-I-19 **(B)**. **(C)** Dual-color FISH in the hendecaploid Sichuan79-I-1 using probes of CP2 (green) and the chromosome 7-specific probe Ss7 (red). **(D)** Dual-color FISH in the hendecaploid Sichuan79-I-1 using the chromosome 5-specific probe Ss5.1 (red) and chromosome 6-specific probe Ss6.2 (green). Ten and eleven signal copies of Ss5.1 and Ss6.2 were observed. **(E)** Dual-color FISH in the hexaploid 2012-46 using CP2 (green) and the chromosome 7-specific probe Ss7 (red). Arrows indicate the six copies of chromosome 2. **(F)** Dual-color FISH in the hexaploid 2012-46 using the chromosome 5-specific probe Ss5.1 (red) and chromosome 6-specific probe Ss6.2 (green). Scale bars, 10 μm.

A genome sequencing study on *S. spontaneum* SES208 proposed an autopolyploid origin by two rounds of whole-genome duplication (Zhang et al., 2018). However, the finding of *S. spontaneum* clones with non-2^*n*^ ploidies, such as decaploid (10x) and dodecaploid (12x), indicates that these clones might be derived from hybridization between clones rather than autopolyploidization. To further examine this hypothesis, we conducted FISH using the CP2 probe in 15 other *S. spontaneum* clones (Table 1, Figure 4, and Supplementary Figure 1). In addition to eight, ten, and twelve copies of chromosome 2, we observed thirteen copies in Guizhou78-II-28 (Figure 4A), Fujian89-I-19 (Figure 4B) and Fujian 87-I-4 (Supplementary Figure 1K). To further confirm ploidy, we conducted FISH using probes specific to chromosomes 5, 6, and 7 (Meng et al., 2020). The results demonstrated 13 copies in these clones (Supplementary Figure 2). In addition, we observed 11 chromosomes with whole-chromosome painting CP2 signals and one chromosome with partial signals in Sichuan79-I-1 (Figure 4C). FISH using chromosomes-specific probes revealed 11 copies of chromosomes 1, 3, 4, 6, and 7 in Sichuan79-I-1, and 10 copies of chromosomes 5 and 8 (Figures 4C,D and Supplementary Figure 3), indicating that it is a hendecaploid with rearrangements in chromosomes 2, 5, and 8. The odd number of ploidies confirmed that certain *S. spontaneum* clones came from hybridization between plants with different ploidies.

**TABLE 1:** The ploidies of *S. spontaneum* clones used in this study.

*S. spontaneum* clones	Ploidies	Locations of plant collection		
		Longitude (E°)	Latitude (N°)	Altitude (m)
2012-46	6x	97°02′	28°27′	1420
Yunnan84-268	8x	98°60′	24°41′	840
Yunnan82-16	8x	97°93′	24°69′	820
Yunnan82-67	8x	100°12′	24°44′	1107
Yunnan82-106	10x	100°79′	22°00′	511
Yunnan82-29	10x	99°10′	25°08′	1800
Yunnan82-110	10x	100°79′	22°00′	511
Yunnan76-III-13	10x	100°50′	21°95′	580
Yunnan76-III-18	10x	101°00′	22°79′	1302
Sichuan92-42	10x	103°93′	29°21′	319
Sichuan79-II-20	10x	108°03′	29°50′	224
Sichuan79-II-18	10x	109°52′	31°06′	287
Sichuan88-16	10x	105°33′	30°31′	800
Sichuan79-I-1	11x	106°15′	30°02′	1000
Sichuan79-II-11	12x	108°03′	30°21′	120
Guangdong30	12x	117°64′	22°95′	150
Fujian89-I-17	12x	/	/	/
Fujian87-I-4	13x	118°16′	25°2′	200
Fujian89-I-19	13x	118°34′	24°49′	55
Guizhou78-II-28	13x	108°53′	25°21′	1100

Interestingly, we observed six copies of chromosome 2 in clone 2012-46 (Figure 4E, arrows). FISH assays using chromosome-specific probes confirmed six copies for each chromosome, indicating that 2012-46 is hexaploid (Figures 4E,F). As we counted the chromosome number 2n = 54, it means that 2012-46 has a basic chromosome number of *x* = 9. This finding confirms the existence of an *S. spontaneum* clone with a basic chromosome number of *x* = 9 (Piperidis and D’Hont, 2020) and may represent an intermediate evolutionary step between the cytotypes of *x* = 10 and *x* = 8.

To examine the morphology between copies of chromosome 2 in each clone, we selected cells without apparent chromosomal morphological distortion for measurement of length in five clones with different ploidies (Supplementary Table 1). The octaploid Yunnan84-268 showed relative lower size coefficient of variation (8.58%) than the clones of decaploid Yunnan82-29 (10.19%), hendecaploid Sichuan79-I-1 (13.60%), dodecaploid Guangdong30 (12.40%), and tridecaploid Fujian87-S1I-4 (11.24%), indicating relative lower size variation in octaploid clone Yunnan84-268 than other clones (Supplementary Table 1).

### Comparative Chromosome Painting Reveals Chromosomal Abnormalities *in Saccharum*

**FIGURE 5:**
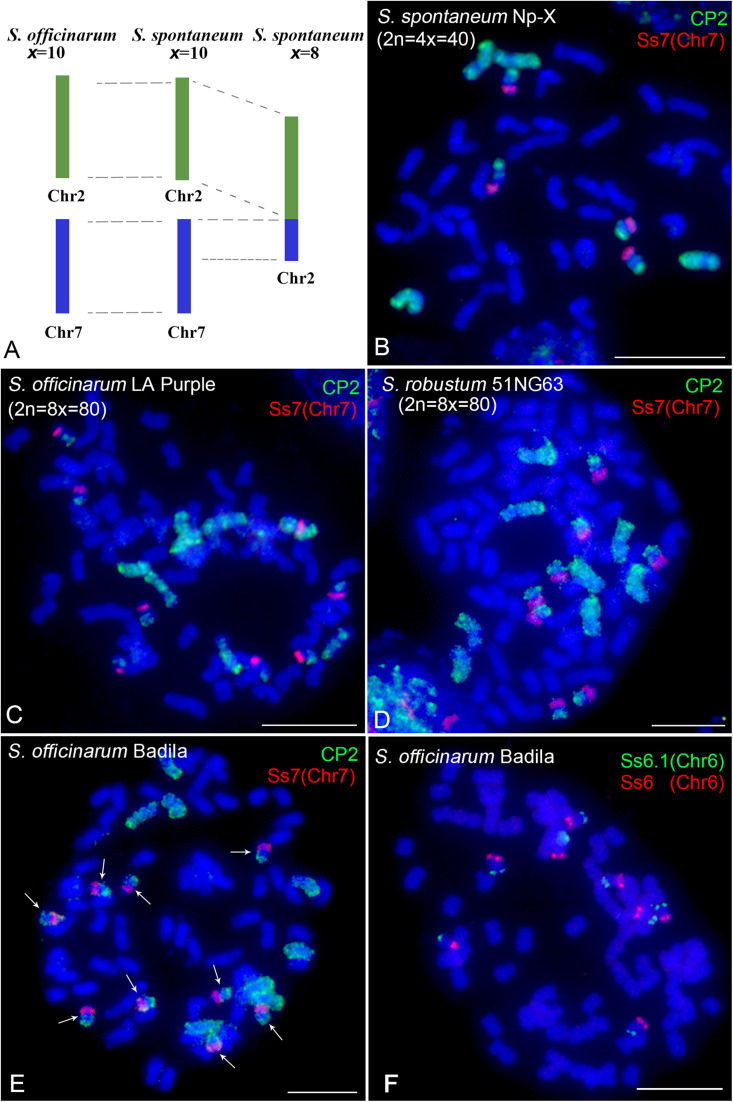
Cross-species chromosome painting assay in *Saccharum*. **(A)** Illustration of chromosome rearrangement between *S. officinarum* and *S. spontaneum*. Chromosome 2 in the *S. spontaneum* clone with the basic chromosome number of *x* = 8 was derived from a fusion of chromosome 2 and a fragment of chromosome 7 in *S. officinarum* and *S. spontaneum* with the basic chromosome number of *x* = 10. **(B–D)** Dual-color FISH using CP2 and the chromosome 7-specific probe Ss7 in *S. spontaneum* Np-X (2n = 4x = 40) **(B)**, *S. officinarum* LA Purple (2n = 8x = 80) **(C)**, *S. robustum* 51NG63 (2n = 8x = 80) **(D)**, and *S. officinarum* Badila **(E)**. Arrows indicate the nine copies of chromosome 7. **(F)** Dual-color FISH using chromosome 6-specific probes Ss6.1 and Ss6 in *S. officinarum* Badila. Scale bars, 10 μm.

Comparative analyses have revealed that basic chromosomes are reduced from *x* = 10 to *x* = 8 in *S. spontaneum* (Zhang et al., 2018; Meng et al., 2020). Chromosome 2 in the *x* = 8 clone originated from the fusion of the entire chromosome 2 and a fragment of chromosome 7 in the *x* = 10 ancestor (Figure 5A). Therefore, we will observe signals covering the whole chromosome 2 and a fragment of chromosome 7 in *x* = 10 *S. spontaneum* Np-X (Figure 5B) using the CP2 probe, which was designed based on *S. spontaneum* with *x* = 8. Similar signal patterns were also observed in the FISH assay using this probe in the *S. officinarum* clone LA Purple (2n = 8x = 80, *x* = 10) and *S. robustum* clone 51NG63 (2n = 8x = 80, *x* = 10) (Figures 5C,D). Notably, we observed nine copies of chromosome 7 in another *S. officinarum* clone Badila (Figure 5E). FISH analyses using another chromosome 7-specific (Ss7) probe (Meng et al., 2020) demonstrated colocalization signals with the CP2 probe, confirming that Badila has nine copies of chromosome 7 (Figure 5E). We then conducted FISH to examine the remaining chromosomes using eight chromosome-specific probes (Meng et al., 2020). The results showed eight consistent copies for each of the eight chromosomes (Figure 5F and Supplementary Figure 4). Taken together, these results revealed that *S. officinarum* Badila is an aneuploid (2n = 81) with an additional copy of chromosome 7.

### Tracing the *S. spontaneum* Chromosome 2- or Chromosome 2-Derived Fragments in Modern Sugarcane Cultivars

**FIGURE 6:**
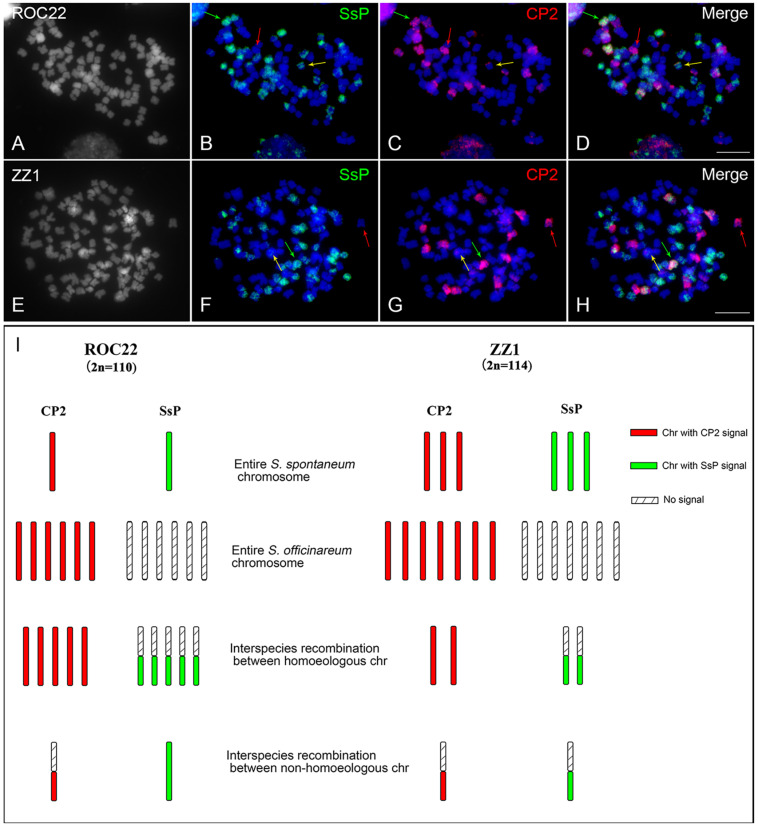
FISH assay using probes of CP2 and *S. spontaneum*-specific repeats in the sugarcane modern cultivars ROC22 and ZZ1. Dual-color FISH assay using CP2 (red) and the *S. spontaneum*-specific probe SsP (green) in sugarcane cultivars ROC22 **(A–C)** and ZZ1 **(D–F)**. **(A,D)** Somatic metaphase chromosomes stained with 4′,6-diamidino-2-phenylindole (DAPI) shown in gray. A representative of entire *S. spontaneum* and entire *S. officinarum* chromosomes are indicated by green and red arrows, respectively. Yellow arrows indicate the translocation chromosome in **(B–D)** and the interspecific recombination chromosome between non-homologous chromosome in **(F–H)**, respectively. Scale bars, 10 μm. **(I)** Diagram illustrating chromosome 2- and chromosome 2-derived chromosomes in cultivars. Each bar represents a chromosome, with colors corresponding to the CP2 or SsP probes used in Figure 6. The chromosomes with CP2 signals are shown on the left of each cultivar. The detection of the SsP signal is shown on the right from the corresponding chromosome with the CP2 signal. Chromosomes with overlapping whole-chromosome signals of CP2 and SsP represent the entire *S. spontaneum* chromosome. Chromosomes with only whole-chromosome CP2 signals represent the entire *S. officinarum* chromosome. Chromosomes with whole-chromosome CP2 signals and regional SsP signals represent interspecific recombination between homoeologous chromosomes. Chromosomes with whole-chromosome SsP signals and regional CP2 signals represent interspecific recombination between non-homoeologous chromosomes.

The purpose of this study was to identify the origin of each chromosome in sugarcane modern cultivars, which derived from interspecific hybridization of *S. officinarum* and *S. spontaneum.* To this end, we examined a dual-probe FISH strategy in which the chromosome 2 painting probe and a recently developed *S. spontaneum*-specific painting (SsP) probe (Huang et al., 2020) were applied to dissect the individual chromosome and its species origin. The SsP probe will generate chromosome painting signals in all chromosomes in *S. spontaneum* but not in *S. officinarum.* Therefore, combed with the individual chromosome painting probe, the chromosome and its species identity will be illustrated in cultivars. According to this strategy, we conducted a FISH assay in the cultivar ROC22, which has been the most planted cultivar in China for more than 20 years. FISH using the CP2 probe revealed 12 chromosomes with an entire painting signal. Among them, one had SsP signal on the entire chromosome, and five had SsP signals on one arm (Figure 6), indicating that there was one entire *S. spontaneum* chromosome 2 and five interspecific recombinations derived from chromosomes 2. In addition, we found a chromosome with a partial CP2 signal but with an entire painting signal of SsP, indicating that it was derived from a translocation event between chromosome 2 and other chromosomes of *S. spontaneum* (Figures 6A–D,I). In another cultivar, ZZ1, we revealed that there were three entire copies of *S. spontaneum* chromosome 2 and two interspecific recombinations derived from chromosomes 2. Interestingly, we observed a chromosome with overlapping signals of SsP and chromosome 2 in a partial region, indicating recombination between *S. spontaneum* chromosome 2 and an unknown *S. officinarum* chromosome (Figures 6E–I). In total, there were seven and six *S. spontaneum*-derived chromosomes in ROC22 and ZZ1, and six and three of them (85.7% and 50%) were involved in chromosome recombination between or within species, respectively (Figure 6I), indicating a high level of chromosome exchange for the introgressive *S. spontaneum* in cultivars.

## Discussion

The large and highly variable chromosome numbers in *Saccharum* present a major challenge for the determination of their chromosomal constitution, structure, and evolution. FISH using rDNA, genomic clone and whole genomic DNA as probes has been applied to decipher the genomic constitution in sugarcane (D’Hont et al., 1995, 1996, 1998; Jannoo et al., 1999; Thumjamras et al., 2016). However, the high noisy signals caused by repetitive sequences from the genomic DNA probes severely limit the development of FISH probe for individual chromosome identification in sugarcane. Chromosome-specific oligo probes based on regional DNA sequences (Regional oligo probes) have recently been developed and used for identification of individual chromosomes in *Saccharum* (Meng et al., 2018, 2020; Piperidis and D’Hont, 2020). However, regional probes can only provide limited information and are incapable of dissecting the chromosomal rearrangement that occurs in the remain regions. Therefore, a CP probe that can trace the entire chromosome is indispensable and has been pursued for sugarcane cytological assays. Compared with the regional probes, CP probes allow investigations at the whole-chromosome level or even the whole-genome level if CP probes are developed for all chromosomes. Unusual chromosomal duplication and chromosome fusion or division can be visualized and confirmed at the individual cell level. A limit of the CP probe developed here is the inability to decipher intrachromosomal rearrangements, such as inversion, deletion, duplication or recombination. The availability of specific oligo probes for a whole chromosome could enable the generation of specific restricted probes (Braz et al., 2018; Albert et al., 2019). Alternating banding paints for a chromosome or for regions of interest could be generated by labeling these specific regional oligos and will eventually allow us to visualize the intrachromosomal rearrangements.

Modern sugarcane cultivars are composed of highly complex genomes derived from *S. officinarum* and *S. spontaneum* hybridization followed by repeated backcrosses to *S. officinarum.* An attempt to assemble the genome has been conducted in sugarcane modern cultivars (∼10 Gb genome size) (Garsmeur et al., 2018). However, the low genome assembly coverage (382 Mb of ∼10 Gb; ∼3.8%) demonstrated the difficulty of dissecting the genome composition of sugarcane cultivars. Combined with the *S. spontaneum*-specific painting probe (Huang et al., 2020), we showed the possibility of uncovering the chromosomal composition in sugarcane cultivars. Here, chromosome 2 can be identified individually by the CP2 probe, and then *S. spontaneum*-derived chromosome 2 or fragments can be distinguished. The wild species *S. spontaneum* is a main donor for sugarcane modern cultivars because it introduces disease resistance and stress tolerance traits in high-sugar-yielding *S. officinarum*. The identification of *S. spontaneum* chromosomes or chromosomal fragments in cultivars will facilitate the identification of such desired trait-bearing chromosomes or chromosomal fragments. To this end, further development of chromosome painting probes for the remaining chromosomes is necessary, and these probes will also permit karyotyping studies on the complex cultivars and diverse material stocks of sugarcane to examine the chromosomal constitution, structure, and evolution in great depth.

In sugarcane modern cultivars, recombination between *S. spontaneum* and *S. officinarum* frequently occurs, leading up to ∼40% of the chromosomes to be derived from interspecific recombinations (Huang et al., 2020). Determining how parental chromosome pairing and recombination produce such descent cultivars remains a fundamental and appealing pursuit for both crop breeders and researchers. Our results in two cultivars confirmed that there is a high level of interspecific recombination in cultivars ROC22 and ZZ1. Intriguingly, we, for the first time, revealed recombination between non-homoeologous chromosomes in ZZ1. The results revealed that interspecific recombination in the cultivar could occur among either homoeologous or non-homoeologous chromosomes, which suggests that there may be a more complex genome structure for sugarcane cultivars than we can imagine. Further study in meiosis using a complete set of CP probes is needed to reinforce these results and will provide us with deep insights into the phenomenon of interspecific recombination, including non-homoeologous chromosome exchange.

*Saccharum spontaneum* shows the highest level of genetic diversity in the *Saccharum* genus (Panje and Babu, 1960; Irvine, 1999; Mary et al., 2006). Different basic chromosome numbers, including *x* = 5 and 8, have been proposed for the wild species *S. spontaneum* (Heinz, 1987). Cytological studies using oligo-FISH have confirmed the existence of *x* = 8 clones (Meng et al., 2018; Piperidis and D’Hont, 2020). Interestingly, clones with basic numbers of *x* = 9 and 10 were also found (Meng et al., 2020; Piperidis and D’Hont, 2020), which presents us with a complete pathway for the evolution of *S. spontaneum* from *x* = 10 to *x* = 8. The clone with *x* = 10 was proposed as an ancestral style of *S. spontaneum*, and clones with *x* = 9 represent an intermediate step between *x* = 10 and *x* = 8. Notably, the clones with *x* = 10 and 9 all came from the northern Indian subcontinent, including Nepal, eastern and western Pakistan, and Sri Lanka (Meng et al., 2020; Piperidis and D’Hont, 2020). In this study, the newly identified *x* = 9 clone was collected from eastern Tibet, China (Yu et al., 2019), close to Yunnan Province, China, but far from the one *x* = 10 clone (found in Nepal). Therefore, the clones with the unusual basic number *x* = 9 or 10 have a wide range of distribution and might have undergone a parallel evolution of genomes in the different regional groups of *S. spontaneum*.

Chromosome number counting has demonstrated that *S. spontaneum* has high levels of diversity with nearly 40 types, including the most frequent types of 64 and 80 (Panje and Babu, 1960; Irvine, 1999). However, the ploidies and evolutionary mechanism of these diverse karyotype types of *S. spontaneum* clones remain a mystery. It is plausible to suppose that all or certain octaploid clones were derived from spontaneous whole-genome duplication from a diploid ancestor as well as the octaploid clone SES208, which has been proposed to have an autopolyploid origin by two rounds of whole-genome duplication based on a genome sequencing study (Zhang et al., 2018). However, our results revealed that the ploidies from the 20 studied clones spanned a wide range, including hexaploid (6x), octaploid (8x), decaploid (10x), hendecaploid (11x), dodecaploid (12x), and tridecaploid (13x) clones. This is the first study to reveal the existence of *S. spontaneum* clones with odd number ploidies. There is no doubt that these two clones are derived from the hybridization between *S. spontaneum* clones with different ploidies. In fact, it is reasonable to suppose that the tetraploid, decaploid, and dodecaploid clones also originated from hybridization between *S. spontaneum* clones. For example, the hexaploid might be derived from a cross between tetraploid and diploid clones followed by one round of whole-genome duplication. If there are two rounds of whole-genome duplication, it will give rise to a dodecaploid clone. The decaploid clone may undergo one round of whole-genome duplication after hybridization between tetraploid and hexaploid clones. The size diversity of the chromosome 2 copies in the clones supports the heterogeneous origin of some *S. spontaneum* clones (Supplementary Table 1). Another potential possibility is that these clones might originate from duplication or deletion on one or a few chromosomal sets. However, this result is unlikely because there is no evidence of spontaneous duplication or deletion occurring only on one or a few chromosomal sets in plants. Taken together, these results indicate that hybridization between clones may frequently occur in *S. spontaneum* and suggest diverse cytotype formation other than autopolyploidization.

## Conclusion

In this study, we developed the first CP probe by designing oligos covering whole chromosome 2 in *S. spontaneum*. FISH assays of 20 *S. spontaneum* clones using this CP2 probe revealed six types of ploidy (i.e., 6x, 8x, 10x, 11x, 12x, and 13x). The finding of odd-ploid *S. spontaneum* clones suggested that certain *S. spontaneum clones* come from hybridization rather than autopolyploidization. In addition, we developed a dual-probe FISH strategy to dissect the individual chromosome and its species origin in sugarcane cultivars. By the dual-probe FISH strategy, we found a high level of interspecific chromosome recombination (>50%) in cultivars ROC22 and ZZ1, indicating frequent interspecific chromosome exchange. Our study confirms that chromosomal painting is a powerful tool for sugarcane genomics research, and further application will provide more useful clues for the genome evolution of complex genus *Saccharum*.

## Data Availability Statement

The datasets presented in this study can be found in online repositories. The names of the repository/repositories and accession number(s) can be found in the article/Supplementary Material.

## Author Contributions

KW and BW acquired financial support and provided overall direction of the project. KW, QW, JH, and ZM conducted the experiments. ZM, HK, QW, GR, BW, and KW analyzed the data and drafted the manuscript. All authors read and approved the manuscript.

## Conflict of Interest

The authors declare that the research was conducted in the absence of any commercial or financial relationships that could be construed as a potential conflict of interest.

## Publisher’s Note

All claims expressed in this article are solely those of the authors and do not necessarily represent those of their affiliated organizations, or those of the publisher, the editors and the reviewers. Any product that may be evaluated in this article, or claim that may be made by its manufacturer, is not guaranteed or endorsed by the publisher.

## Footnotes

1
https://github.com/forrestzhang/Chorus

2
https://www.ncbi.nlm.nih.gov/genome/54791

## Supplementary Material

Supplementary Figure 1 FISH mapping with the CP2 probe in *S. spontaneum* clones. FISH assays using the CP2 probe in 11 *S. spontaneum* clones, Yunnan82-16 **(A)**, Yunnan82-67 **(B)**, Yunnan82-110 **(C)**, Yunnan76-III-13 **(D)**, Yunnan76-III-18 **(E)**, Sichuan92-42 **(F)**, Sichuan79-II-20 **(G)**, Sichuan79-II-18 **(H)**, Sichuan88-16 **(I)**, Sichuan79-II-11 **(J)**, and Fujian87-I-4 **(K)**. The ploidy is indicated on the corresponding clones. Scale bars, 10 μm.

Supplementary Figure 2 FISH mapping using chromosome-specific probes in the three tridecaploid *S. spontaneum* clones. Three chromosome-specific probes, Ss5.1, Ss6.2, and Ss7, which are located on chromosomes 5, 6, and 7, respectively, were hybridized to the three *S. spontaneum* clones Fujian89-I-19 **(A,B)**, Fujian87-I-4 **(C,D)**, and Guizhou78-II-28 **(E,F)**. Thirteen copies of signals were observed from each probe. Scale bars, 10 μm.

Supplementary Figure 3 FISH mapping using chromosome-specific probes in *S. spontaneum* Sichuan79-I-1. Probes specific to chromosomes 1 (Ss1), 3 (Ss3), 4 (Ss4), and 8 (Ss8.1) were hybridized in Sichuan79-I-1. Eleven signal copies from probes of Ss1 **(A)**, Ss3 **(B)**, and Ss4 **(C)**, and ten copies from Ss8.1 **(D)** were observed. The probes used in FISH is indicated in the corresponding panel. Scale bars, 10 μm.

Supplementary Figure 4 FISH mapping using chromosome-specific probes in *S. officinarum* Badila. Probes specific to chromosomes 1 **(A)**, 3–5 **(B–D)**, and 8–10 **(E–G)** were hybridized in Badila. Eight signal copies for each probe were observed. The probe used in FISH is indicated in the corresponding panel. Scale bars, 10 μm.

Supplementary Data Sheet 1 The oligo sequence for CP2 probe.
