# Supplementary figures and images for: Chromosome Painting Provides Insights Into the Genome Structure and Evolution of Sugarcane
Source: Front Plant Sci. 2021 Aug 27;12:731664. doi: 10.3389/fpls.2021.731664 (PMC8429501; doi:10.3389/fpls.2021.731664)

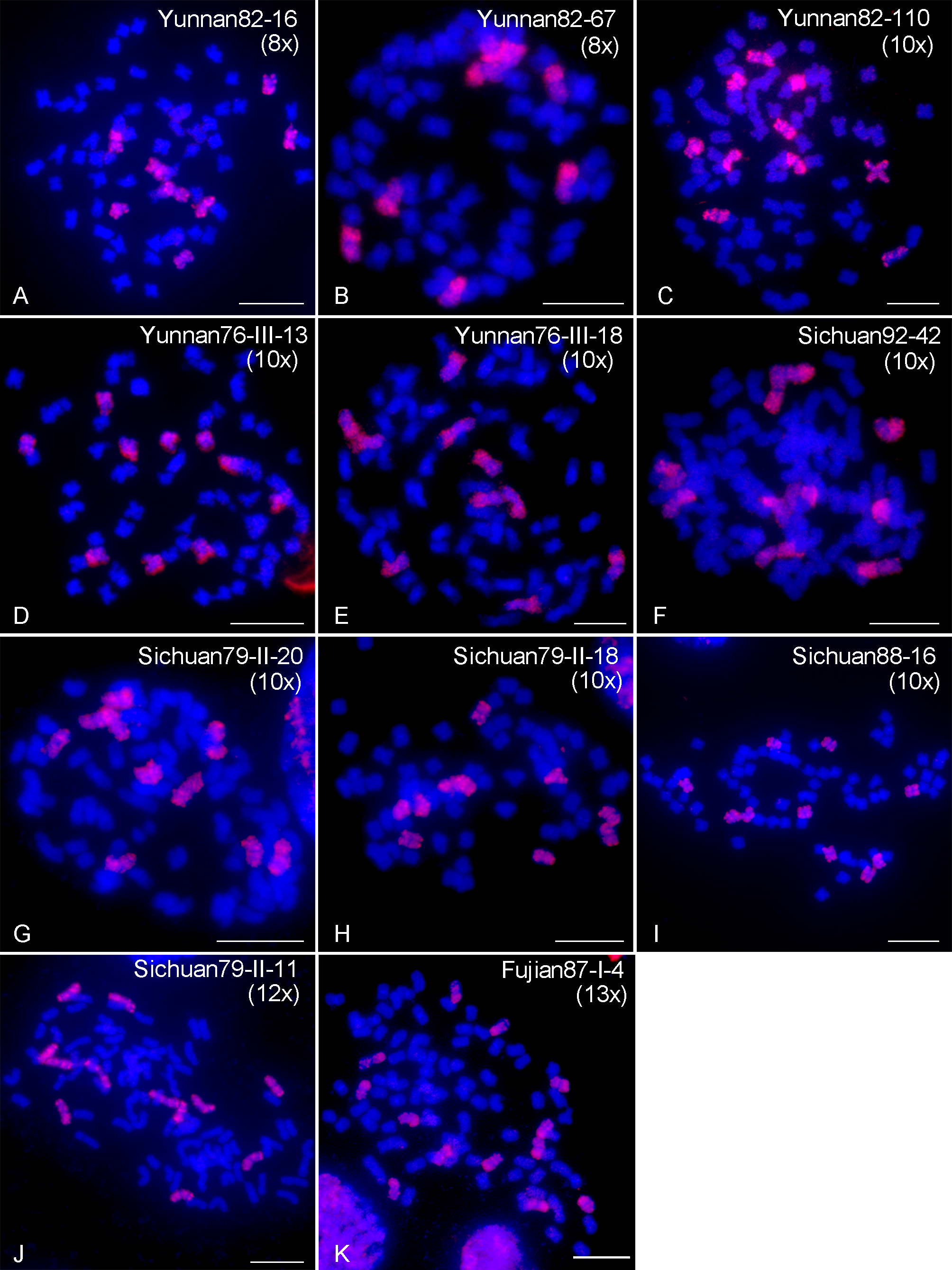

Supplement: Supplementary Figure 1 — FISH mapping with the CP2 probe in S. spontaneum clones. FISH assays using the CP2 probe in 11 S. spontaneum clones, Yunnan82-16 (A), Yunnan82-67 (B), Yunnan82-110 (C), Yunnan76-III-13 (D), Yunnan76-III-18 (E), Sichuan92-42 (F), Sichuan79-II-20 (G), Sichuan79-II-18 (H), Sichuan88-16 (I), Sichuan79-II-11 (J), and Fujian87-I-4 (K). The ploidy is indicated on the corresponding clones. Scale bars, 10 μm. [file Image_1.TIF]

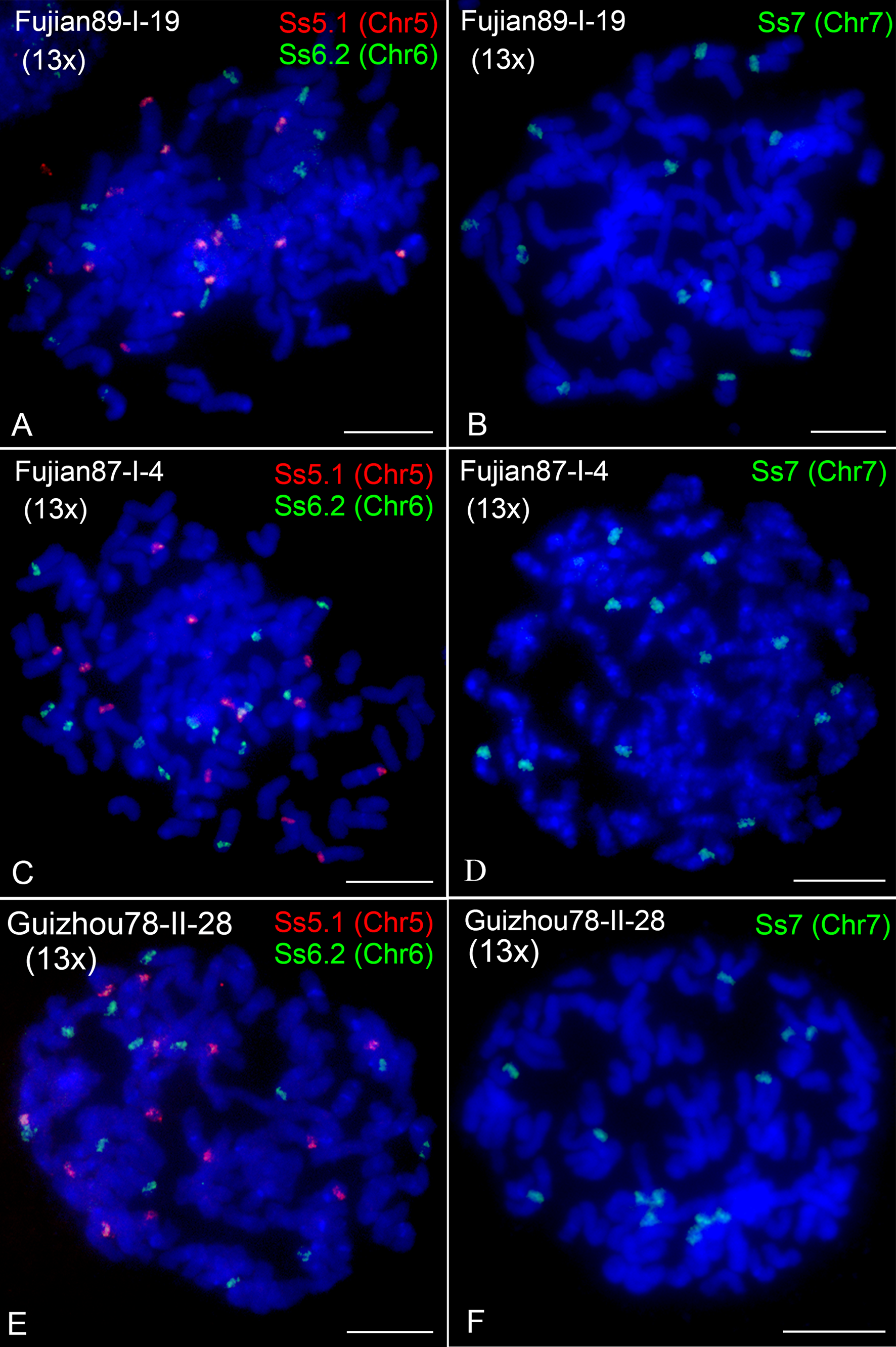

Supplement: Supplementary Figure 2 — FISH mapping using chromosome-specific probes in the three tridecaploid S. spontaneum clones. Three chromosome-specific probes, Ss5.1, Ss6.2, and Ss7, which are located on chromosomes 5, 6, and 7, respectively, were hybridized to the three S. spontaneum clones Fujian89-I-19 (A,B), Fujian87-I-4 (C,D), and Guizhou78-II-28 (E,F). Thirteen copies of signals were observed from each probe. Scale bars, 10 μm. [file Image_2.TIF]

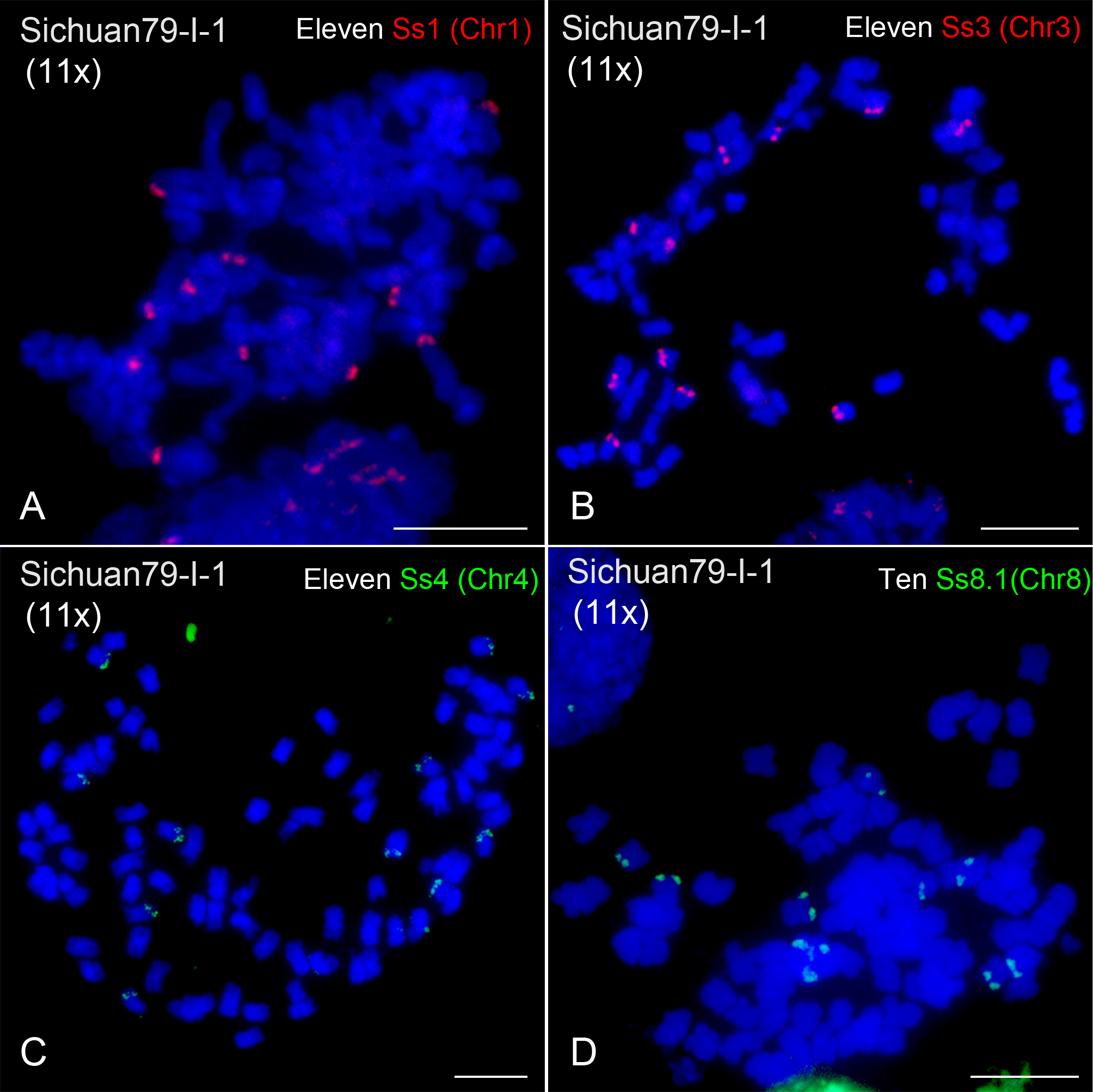

Supplement: Supplementary Figure 3 — FISH mapping using chromosome-specific probes in S. spontaneum Sichuan79-I-1. Probes specific to chromosomes 1 (Ss1), 3 (Ss3), 4 (Ss4), and 8 (Ss8.1) were hybridized in Sichuan79-I-1. Eleven signal copies from probes of Ss1 (A), Ss3 (B), and Ss4 (C), and ten copies from Ss8.1 (D) were observed. The probes used in FISH is indicated in the corresponding panel. Scale bars, 10 μm. [file Image_3.TIF]

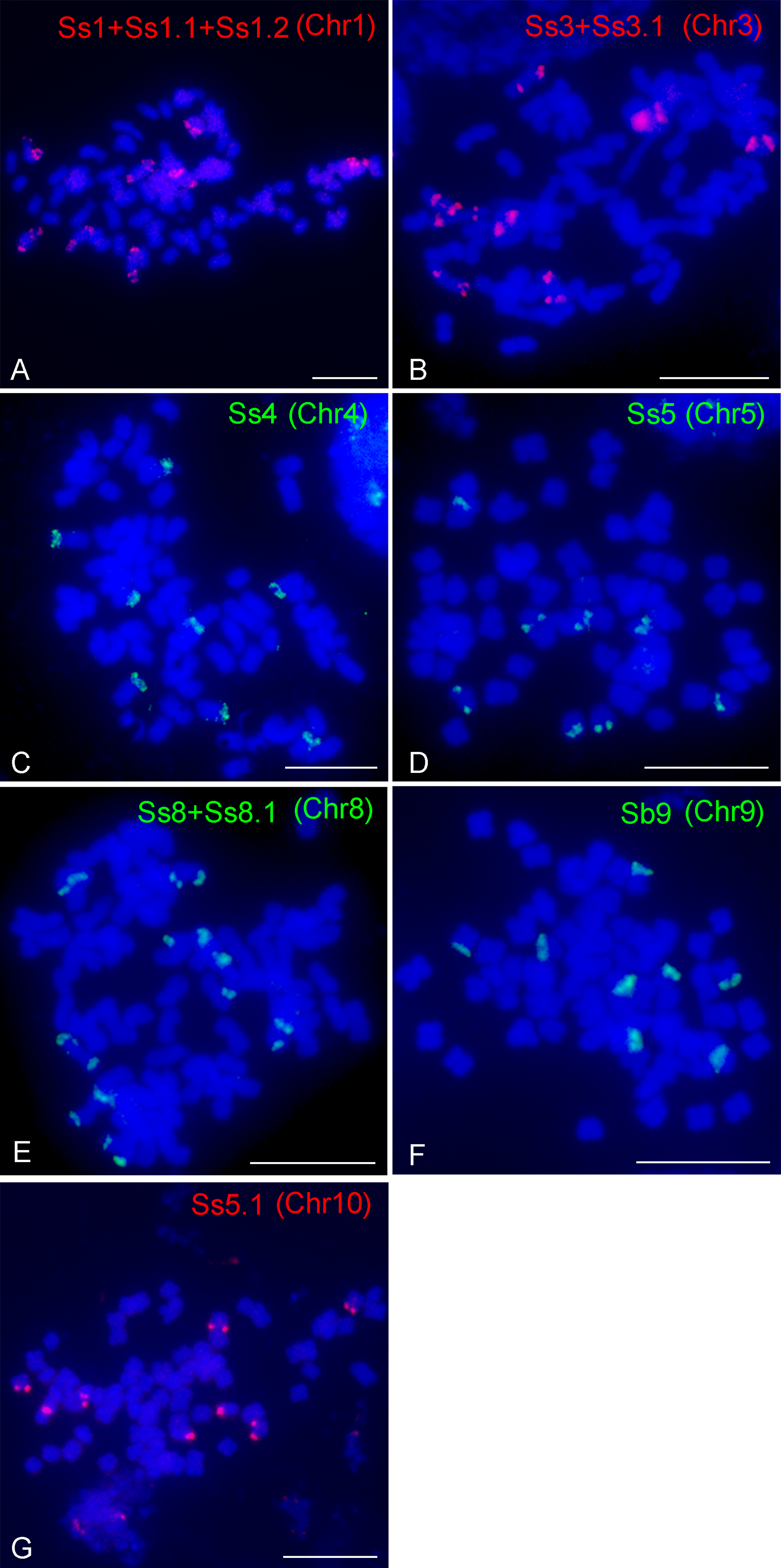

Supplement: Supplementary Figure 4 — FISH mapping using chromosome-specific probes in S. officinarum Badila. Probes specific to chromosomes 1 (A), 3–5 (B–D), and 8–10 (E–G) were hybridized in Badila. Eight signal copies for each probe were observed. The probe used in FISH is indicated in the corresponding panel. Scale bars, 10 μm. [file Image_4.TIF]
