# Supplementary material for: Chromosome Painting Provides Insights Into the Genome Structure and Evolution of Sugarcane
Source: Front Plant Sci. 2021 Aug 27;12:731664. doi: 10.3389/fpls.2021.731664 (PMC8429501; doi:10.3389/fpls.2021.731664)
Supplement: Supplementary file 6 [file Table_1.DOCX]

**Table S1.** **Coefficient of variation between chromosome 2 homologs in each *S. spontaneum* clone**

| *S. spontaneum* clones | C.V (%) | n^a^ |
| --- | --- | --- |
| Yunnan84-268 (8x) | 8.58 | 10 |
| Yunnan82-29 (10x) | 10.19 | 10 |
| Guangdong30 (12x) | 12.40 | 10 |
| Fujian87-I-4 (13x) | 11.24 | 7 |
| Sichuan79-I-1 (11x) | 13.60 | 8 |

^a^Number of cells analyzed.
